# Amyloid quantification in the oldest-old: selecting regions for optimizing correspondence between postmortem pathology and amyloid PET

**DOI:** 10.1186/s40478-025-02198-3

**Published:** 2025-12-01

**Authors:** Jiaxin Yu, Davis C. Woodworth, Evan Fletcher, Dana E. Greenia, Syed Bukhari, Thomas J. Montine, Maria M. Corrada, Claudia H. Kawas, Charles DeCarli, S. Ahmad Sajjadi, Tianchen Qian

**Affiliations:** 1 Department of Statistics, University of California, Irvine, CA 92697 USA; 2 Department of Neurology, University of California, Irvine, CA 92868 USA; 3 Institute for Memory Impairments and Neurological Disorders, University of California, Irvine, CA 92697 USA; 4 Imaging of Dementia and Aging Laboratory, Department of Neurology, University of California, Davis, CA 95817 USA; 5 Department of Pathology, Stanford University, Palo Alto, CA 94305 USA; 6 Department of Epidemiology and Biostatistics, University of California, Irvine, CA 92697 USA; 7 Department of Neurobiology and Behavior, University of California, Irvine, CA 92697 USA

**Keywords:** Amyloid positron emission tomography, Neuropathology, Oldest-old, Standardized uptake value ratios

## Abstract

Positron emission tomography (PET) is the current gold standard for assessing amyloid burden in vivo and is often quantified using standardized uptake value ratios (SUVRs). We evaluated the performance of four SUVR calculation methods for predicting amyloid deposition at autopsy in a group of oldest-old participants. We analyzed data from 165 participants from *The 90 + Study* with both florbetapir PET and postmortem assessments. PET scans were re-aligned to an older age template using a custom MRI-free pipeline. SUVRs were computed from two target regions—combined posterior cingulate and precuneus (PC^2^) and an established cortical summary region—and two reference regions—white matter (WM) and cerebellar gray matter. Their predictive performance for amyloid beta positivity (Thal phase ≥ 3) and neuritic plaque positivity (moderate or frequent CERAD score) was evaluated using receiver operating characteristic analyses, including DeLong’s test for comparisons. Participants had a mean (± standard deviation) age at PET of 94 ± 3 years, and age at death of 97 ± 4 years. Most participants had amyloid at autopsy: 113 (68%) were amyloid beta positive, and 104 (63%) were neuritic plaque positive. The best performing SUVR was the PC^2^+WM, yielding an area under the curve (AUC) of 0.84 (95% CI 0.78–0.90) for amyloid beta and 0.82 (95% CI 0.76–0.89) for neuritic plaque, with an optimal cutoff of 0.77 for both outcomes. Reference and target region selection was more consequential for predicting amyloid beta positivity, with the WM consistently outperforming the cerebellar gray matter as a reference region for both target regions, while the PC^2^ significantly outperformed the cortical summary region for the cerebellar gray matter reference (*P* < 0.001) but only trended for significance for the WM reference (*P* = 0.069, DeLong’s test). Our findings suggest that using a WM reference and a limited cortical target region may improve PET-neuropathology correspondence, especially in older cohorts.

**Supplementary Information:**

The online version contains supplementary material available at 10.1186/s40478-025-02198-3.

## Introduction

Accumulation of amyloid β plaques in the brain is an essential characteristic of Alzheimer’s disease (AD). The severity of amyloid burden predicts subsequent cognitive decline, and biomarkers of amyloid are cornerstones of research and clinical frameworks for AD [1, 2]. Furthermore, the advent of amyloid-targeting therapies has placed new importance on in vivo amyloid measures that will now be used for treatment decisions [3].Thus, biomarkers of amyloid are a critical tool for early detection, disease monitoring, and treatment of AD [4]. Among the various biomarkers presently available, positron emission tomography (PET) imaging is the gold standard for in vivo assessment of amyloid burden [5]. Quantitative analysis of amyloid burden in AD patients is often reported using standardized uptake value ratios (SUVRs), which are calculated by normalizing amyloid β PET tracer uptake values from a target brain region of interest (ROI) to a reference region that is presumed to be minimally affected by amyloid deposition [6–9].

The accuracy of SUVR-based amyloid quantification is highly sensitive to the selection of ROI and reference region [10, 11]. Extensive studies have examined the differential effects of using various reference regions, including cerebellar gray matter, whole cerebellum, and subcortical white matter, noting that each choice can affect both the sensitive detection of amyloid accumulation and the dynamic range of SUVR values [7, 9, 12–14]. The choice of target ROI is typically based on brain regions that are prone to the deposition of amyloid β plaques, including the frontal, temporal, and cingulate cortices. However, research on amyloid quantification has predominantly focused on younger older adults (e.g., individuals in their 70–80 s) [9, 15]. The oldest-old population, those 90 years on older, is rapidly increasing [16, 17], and neuroimaging studies in this group remain relatively scarce [18, 19]. This gap is particularly concerning because advanced atrophy, multimorbidity, and contraindications for MRI are common among the oldest-old, potentially complicating standard imaging protocols and SUVR reliability. Some studies suggest that cerebellar amyloid deposition may occur in older populations, which highlights the need to explore alternatives reference regions such as white matter [7, 9, 15]. In the oldest-old, amyloid burden continues to be predictive of future dementia [19–21], and accurate quantification of amyloid may be more important in this age group given an increased incidence of dementia as well as increased frequency of non-AD neuropathologic changes [22–24].

This study addresses these critical gaps by systematically evaluating multiple SUVR measures against the gold standard of amyloid neuropathology from autopsy in a cohort of oldest-old participants. Specifically, we focused on two potential reference regions (cerebellar gray matter or white matter) and two potential target regions (a standard cortical summary region and a selected, more limited, cortical region of the posterior cingulate and precuneus) and we examined which combination of these best aligned the amyloid PET data with postmortem evidence of amyloid β. Furthermore, we sought to establish optimal cutoffs for these SUVRs based on amyloid neuropathology at autopsy.

## Methods

### Study population

We used data from participants of *The 90 + Study* who had both florbetapir PET and postmortem neuropathological assessments. *The 90 + Study* is a study on aging and dementia in participants aged 90 years or older based in southern California, with participants originally recruited from survivors from the Leisure World Cohort Study (LWCS) in 2003 [25], but with subsequent expanded recruitment targeting individuals without contraindications for imaging and emphasized brain donation at autopsy [26].

### Determination of dementia

Clinical diagnosis of dementia was established according to Diagnostic and Statistical Manual of Mental Disorders 4th edition (DSM-IV), through a multidisciplinary consensus diagnostic conference conducted after a participant’s death, with conferees blinded to PET biomarker data and pathological findings. All clinical and neuropsychological data obtained through in-person, longitudinal assessments on the participant was used in determination of dementia status. This included neuropsychological test scores, neurological examination, information collected from informants, available medical records, videos containing semi-structured interviews about their daily life and questions to test their memory, as well as brief examination of their gait from the time of visits, and death certificates.

### APOE genotyping

DNA samples for apolipoprotein E (APOE) genotyping were obtained through either a cheek swab or a blood draw. Participants were classified as APOE ε4 carriers if they had at least one ε4 allele and as noncarriers if they did not possess an ε4 allele.

### Amyloid PET acquisition and preprocessing

Participants were scanned on an ECAT high-resolution research tomograph PET scanner (HRRT, CTI/Siemens, Knoxville, TN) using florbetapir. Two 5-min emission scans were acquired 50 min after injection of 10 mCi of the radiotracer, followed by a 5-min transmission scan for attenuation correction. PET scans were reconstructed using 4 iterations and 16 subsets of the 3D ordinary Poisson ordered subset expectation maximization (OP-OSEM) algorithm with 5-mm smoothing. Because many participants in the cohort lacked an accompanying MRI scan, and because many amongst the oldest-old population are excluded from having MRIs (because of pacemakers or surgical devices or other physical restrictions), we processed the amyloid PET scans using an MRI-free pipeline. The MRI-free pipeline was developed following principles described by Landau et al. (2022) [27]. We used a PET template created by Landau and colleagues at the University of California, Berkeley and linearly aligned the generic template to a common space (minimal deformation target [5], (MDT3)) developed by the Imaging of Dementia and Aging (IDeA) laboratory at the University of California, Davis. We then further warped it to MDT3 using B-spline transformation [6] custom software used in IDeA laboratory neuroimaging processing pipelines. These aligned templates served as targets for first linearly and then nonlinearly aligning each native PET image. Visual quality control was performed to ensure satisfactory alignment of all deformed PET images into the common space. After motion correction and averaging, the amyloid scans were diffeomorphically aligned to an amyloid PET atlas, constructed from amyloid scans from older individuals in the age range of 60 + with available MRI for accurate PET–MRI registration [27]. We performed visual quality control (QC) to insure satisfactory matches of all deformed PET images into common space. The amyloid images were further smoothed with a 3D 6-mm Gaussian kernel.

### SUVR calculation

While large sections of cortical tissue (e.g. cortical summary including frontal, temporal, parietal, cingulate regions, or entire cortex) are usually used as the target region for SUVR calculation, these regions can lead to less robust estimates of amyloid burden in the oldest-old because of the high degree of brain atrophy in this population. Previous research by our group has explored more limited cortical regions and identified the posterior cingulate and precuneus (PC^2^) as suitable regions for SUVR calculation [18, 21, 28]. Amyloid deposition appears early in PC^2^, and it remains relatively preserved until the late stage of AD, even when global atrophy is considerable in the oldest-old. Moreover, PC^2^ yielded the highest sensitivity and specificity for amyloid status at autopsy in previous preliminary data-driven work [28]. For these reasons PC^2^ was included as one of the target ROIs in this study.

Four SUVRs were generated by using combinations of two target regions and two reference regions. The target regions were the PC^2^ region and a pre-existing cortical summary region composed of frontal, anterior/posterior cingulate, lateral parietal, and lateral temporal regions [27, 29]. The reference regions were eroded subcortical white matter (WM) and cerebellar gray matter (Cbllm GM). Each of these regions was obtained from a brain region atlas from the IDeA lab constructed using scans from participants older than 60 years [30]. The eroded WM region was obtained by eroding the cerebral white matter mask by 1 mm. The average amyloid uptake from the diffeomorphically aligned amyloid images within each of these regions was then used to calculate the SUVRs.

### Neuropathological assessment

Participants were assessed neuropathologically using standard guidelines [31], with pathologists blinded to clinical, imaging, and fluid biomarker results. Methods for detection and quantification of amyloid in brain sections were defined using two neuropathological outcomes: amyloid beta positivity and neuritic plaque positivity. Amyloid beta score was characterized by Thal phases, with a ranked level (A0 = none, A1 = Thal phase 1 or 2, A2 = Thal phase 3, A3 = Thal phase 4 or 5) [32, 33]. Neuritic plaque score was characterized by the Consortium to Establish a Registry for Alzheimer’s Disease (CERAD) scoring system, which has a semiquantitative rating (C0 = none, C1 = sparse, C2 = moderate, or C3 = frequent neuritic plaques) [33, 34]. An A-score of 2 or 3 was defined as amyloid beta positive, and a C-score of 2 or 3 was defined as neuritic plaque positive.

### Statistical analyses

We evaluated the predictive performance of the four SUVRs for the two binary outcomes of amyloid beta positivity and neuritic plaque positivity. Receiver operating characteristic (ROC) analysis was conducted to assess the discrimination ability of each SUVR, quantified by the area under the curve (AUC). We further performed one-sided DeLong tests to formally compare the AUC of the SUVR. The optimal cutoff for each SUVR is the cutoff value that maximizes the Youden index, i.e., the sum of sensitivity and specificity. ROC analysis was performed in R using pROC package [35]. For the optimal cutoff for each SUVR, sensitivity, specificity, and accuracy (defined as the proportion of correct classifications) were calculated and reported, and their confidence intervals were obtained by using the Clopper and Pearson procedure [36].

We further conducted multiple sensitivity analyses to evaluate the robustness of the findings. First, we incorporated additional demographic covariates, including sex, age at PET, education level, and APOE genotype into the ROC analysis to account for potential confounding factors and assess whether the inclusion of these variables impacted the predictive performance of the SUVRs. Second, in addition to the demographic factors, we included the time interval from PET to death, and an interaction between this time interval and amyloid PET burden. This interaction term examines whether the time interval between PET and death may influence amyloid deposition and its correspondence with neuropathology. Third, to ensure that the results were consistent across participants with different time intervals from PET to death, we ran the ROC analysis of the SUVR-only model in two sub-cohorts: participants who underwent PET scans within five years of death and those who underwent PET scans within three years of death. The five-year and three-year cutoffs were determined based on the histogram of time intervals from PET to death (Supplementary Fig. 1). Finally, we evaluated the robustness of our findings using an alternative dichotomization method for defining amyloid positivity. Specifically, we redefined amyloid beta positivity as A-score of 3 and neuritic plaque positivity as C-score of 3 and repeated the ROC analyses to ensure consistency of results under different definitions.

## Results

Table 1 presents the demographic and clinical characteristics of the 165 participants included in this study, stratified by amyloid beta positivity and neuritic plaque positivity. 113 (68%) of the participants were amyloid beta positive, and 104 (63%) were neuritic plaque positive; 96 (58.2%) were positive and 44 (26.7%) were negative for both scores. Of the 165 participants, 46 (28%) had dementia at death, with a significantly higher prevalence in those with the presence of amyloid beta (*p* = 0.040) and presence of neuritic plaque (*p* < 0.001). The mean age at PET imaging was 94.2 years (SD 3.2), and mean age at death of 97.4 years (SD 3.6). 136 (82.4%) of the participants had a PET scan within 5 years before death, and 84 (50.9%) of the participants had a PET scan within 3 years before death (Supplementary Fig. 1). 90 (55%) of the participants had a college degree or higher, 98 (59%) were women, and 24 (16%) APOE ε4 carriers.

**Table 1:** Characteristics of the participants stratified by amyloid beta positivity and neuritic plaque positivity

	Whole group (*N* = 165)	Amyloid beta score			Neuritic plaque score		
		Negative (A0/A1) (*N* = 52)	Positive (A2/A3) (*N* = 113)	*P* value	Negative (C0/C1) (*N* = 61)	Positive (C2/C3) (*N* = 104)	*P* value
Age at PET	94.23 (3.20)	93.75 (3.43)	94.44 (3.08)	0.08	93.73 (3.46)	94.51 (3.01)	0.04
Age at death	97.4 (3.6)	96.8 (3.8)	97.6 (3.5)	0.11	97.0 (4.2)	97.6 (3.2)	0.14
Female	98 (59%)	28 (54%)	70 (62%)	0.3	32 (52%)	66 (63%)	0.2
College graduate	90 (55%)	24 (46%)	66 (58%)	0.14	30 (49%)	60 (58%)	0.3
APOE $\:\varepsilon 4$+^a^	24 (16%)	3 (6.5%)	21 (21%)	< 0.001	4 (7.4%)	20 (21%)	0.028
Diagnosis at death				0.05			< 0.001
CN	57 (35%)	24 (46%)	33 (29%)		29 (48%)	28 (27%)	
CIND	62 (38%)	19 (37%)	43 (38%)		25 (41%)	37 (36%)	
Dementia	46 (28%)	9 (17%)	37 (33%)		7 (11%)	39 (38%)	
Amyloid beta score							< 0.001
Negative (A0/A1)	52 (32%)				44 (72%)	8 (7.7%)	
Positive (A2/A3)	113 (68%)				17 (28%)	96 (92%)	
Neuritic plaque score				< 0.001			
Negative (C0/C1)	61 (37%)	44 (85%)	17 (15%)				
Positive (C2/C3)	104 (63%)	8 (15%)	96 (85%)				

An A score of 2 or 3 (A2 or A3) is defined as amyloid beta positive; A C score of 2 or 3 (C2 or C3) is defined as neuritic plaque positive. *P* values were obtained from *t* test or Fisher’s exact test.

N = number of participants; A0 = no amyloid beta; A1 = Thal phases 1 or 2; A2 = Thal phase 3; A3 = Thal phases 4 or 5; C0 = no neuritic plaques; C1 = a sparse CERAD score, C2 = a moderate CERAD score; C3 = a frequent CERAD score; CN = cognitively normal; CIND = cognitive impairment no dementia.

*APOE* $\:\epsilon\:4$+^a^ 17 participants did not have available APOE ε4 data.

**Fig. 1:**
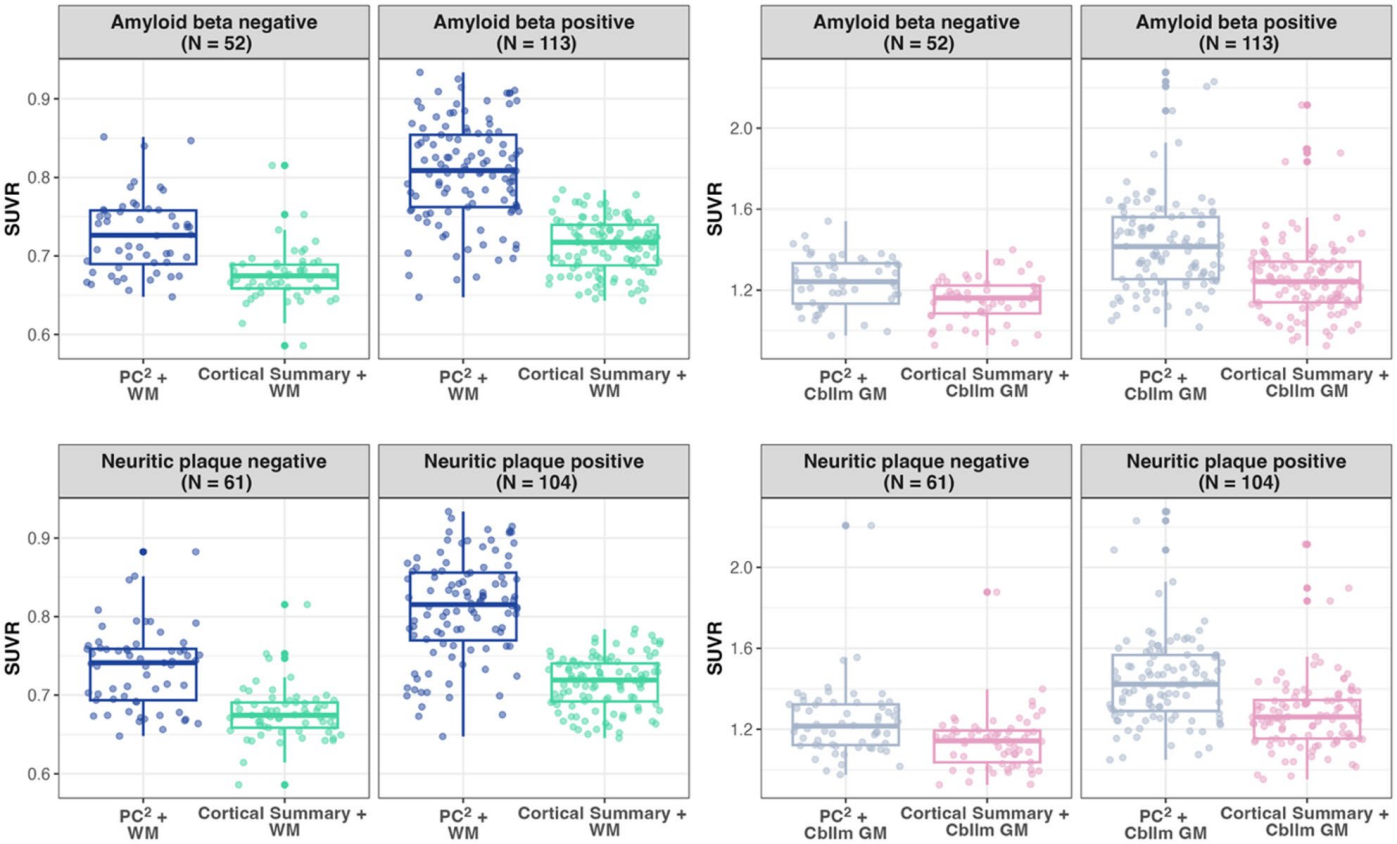
Boxplots of four SUVRs by levels of dichotomized amyloid beta positivity and neuritic plaque positivity. Each color represents a different SUVR with their names specified below. Abbreviations: PC^2^ = posterior cingulate and precuneus; cortical summary = a composite cortical summary region composed of frontal, anterior/posterior cingulate, lateral parietal, and lateral temporal regions; WM = eroded white matter; Cbllm GM = cerebellar gray matter

Figure 1 shows the distributions of the four SUVRs stratified by amyloid beta positivity and neuritic plaque positivity. Across all combinations, SUVRs were consistently higher in the amyloid beta positive and neuritic plaque positive groups. Given the same reference region, PC^2^ SUVRs were on average higher than cortical summary based SUVRs. As shown in the correlation heatmap in Supplementary Fig. 2, SUVRs based on the same reference region demonstrated strong correlations: SUVRs from PC^2^ + WM and cortical summary + WM had a correlation of 0.78, and SUVRs from PC^2^ + Cbllm GM and cortical summary + Cbllm GM had a correlation of 0.93. In contrast, SUVRs based on the same target region but different reference regions displayed substantially lower correlations: PC^2^ + WM and PC^2^ + Cbllm had a correlation of 0.47, and cortical summary + WM and cortical summary + Cbllm GM had a correlation of 0.17.

### SUVR comparisons

**Fig. 2:**
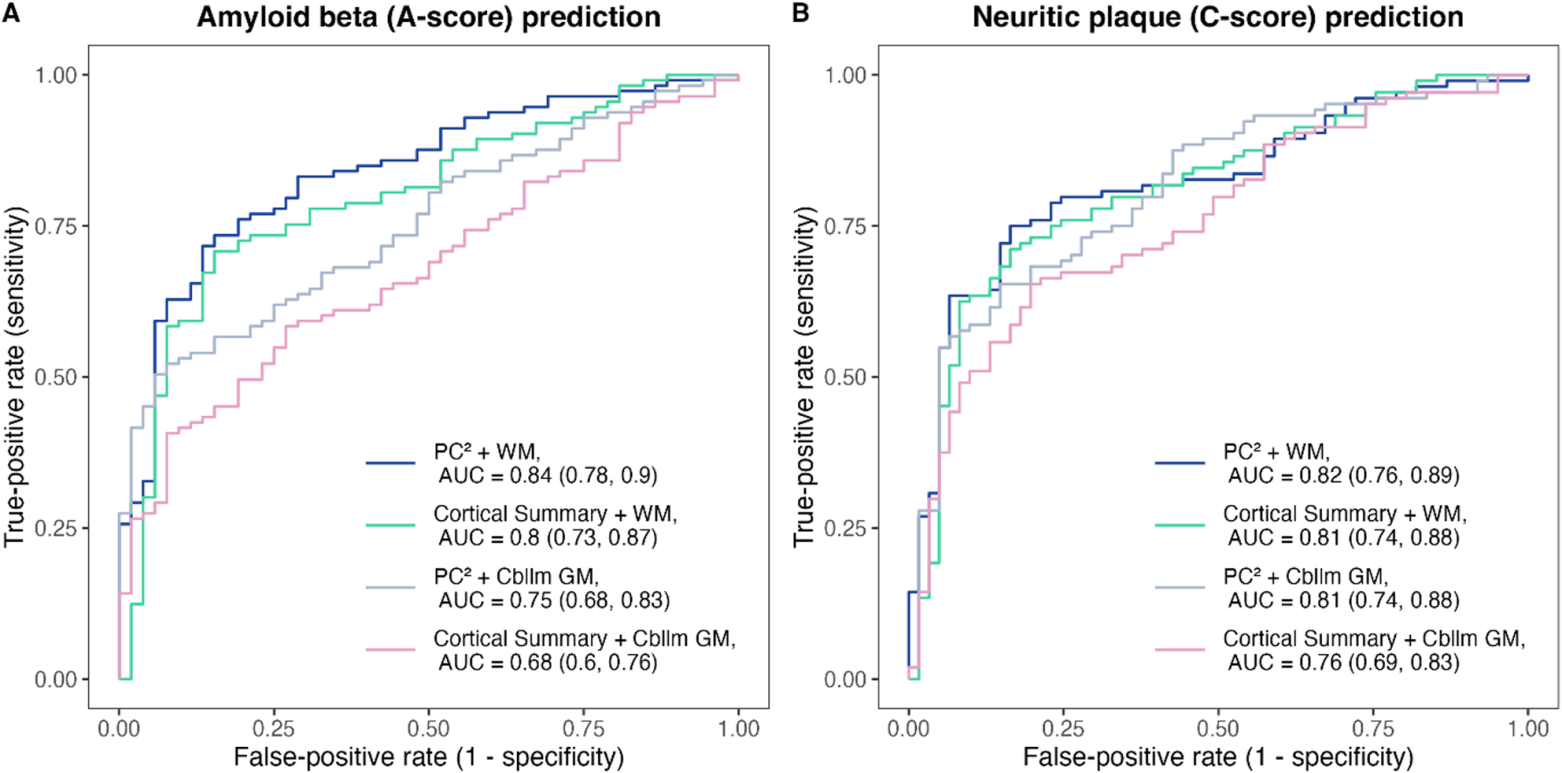
Receiver operating characteristic curves for amyloid beta prediction (**A**) neuritic plaque prediction (**B**) of four SUVRs calculated using two different target regions and two different reference regions. The numbers in parentheses represent 95% confidence intervals of the AUCs. The highest AUC for predicting both amyloid outcomes was the combination of using posterior cingulate and precuneus as target region and white matter as reference region. Abbreviations: AUC = area under the receiver operating characteristic curve; PC^2^ = posterior cingulate and precuneus; cortical summary = a composite cortical summary region composed of frontal, anterior/posterior cingulate, lateral parietal, and lateral temporal regions; WM = eroded white matter; Cbllm GM = cerebellar gray matter

In the ROC analyses for predicting amyloid beta positivity (Fig. 2, Panel A), the two SUVRs using WM as the reference region demonstrated better performance, with PC^2^ + WM achieving the highest AUC of 0.84 (95% CI 0.78–0.90) and cortical summary + WM following with an AUC of 0.80 (95% CI 0.73–0.87). The difference between these two only trended towards significance based on the DeLong test (*p* = 0.069). In contrast, the two SUVRs using cerebellar GM as the reference region showed lower predictive performance, with PC^2^ + Cbllm GM yielding an AUC of 0.75 (95% CI 0.68–0.87) and cortical summary + Cbllm GM yielding an AUC of 0.68 (95% CI 0.60–0.76). Based on Delong’s test, the combination of PC^2^ + Cbllm GM outperforms cortical summary + Cbllm GM (*p* < 0.001). When using PC^2^ as the target region, PC^2^ + WM outperforms PC^2^ + Cbllm GM (*p* = 0.024). When using cortical summary as the target region, WM still outperforms Cbllm GM (*p* = 0.018).

In the ROC analyses for predicting neuritic plaque positivity (Fig. 2, Panel B), similar patterns were observed: PC^2^ + WM achieved an AUC of 0.82 (95% CI 0.76–0.89), cortical summary + WM an AUC of 0.81 (95% CI 0.74–0.88), PC^2^ + Cbllm GM an AUC of 0.81 (95% CI 0.74–0.88), and cortical summary + Cbllm GM an AUC of 0.76 (95% CI 0.69–0.83). DeLong tests indicated a significant difference only for the comparison between PC^2^ + Cbllm GM and cortical summary + Cbllm GM (*p* = 0.0028).

### SUVR optimal cutoffs

When determining the optimal threshold for amyloid beta positivity (Table 2), the SUVR cutoff of 0.77 in PC^2^ + WM achieved the highest Youden index, with sensitivity of 0.72 (95% CI 0.62–0.80) and specificity of 0.87 (95% CI 0.74–0.94). For cortical summary + WM, the optimal cutoff of 0.69 yielded a sensitivity of 0.71 (95% CI 0.61–0.79) and a specificity of 0.85 (95% CI 0.72–0.93). When using cerebellar gray matter as reference, PC^2^ + Cbllm GM showed optimal performance at a cutoff of 1.41, with sensitivity of 0.50 (95% CI 0.41–0.60) and specificity of 0.94 (95% CI 0.84–0.99). cortical summary + Cbllm GM achieved optimal performance at a SUVR cutoff of 1.29, with sensitivity of 0.41 (95% CI 0.32–0.50) and specificity of 0.92 (95% CI 0.81–0.98).

**Table 2:** Optimal cutoffs of the four SUVRs in classifying amyloid beta positivity and neuritic plaque positivity

Reference region	Target region	Optimal cutoff	Sensitivity	Specificity	Accuracy
Amyloid beta					
WM	PC^2^	0.77	0.72 (0.62, 0.8)	0.87 (0.74, 0.94)	0.76 (0.69, 0.83)
	Cortical Summary	0.69	0.71 (0.61, 0.79)	0.85 (0.72, 0.93)	0.75 (0.68, 0.82)
Cbllm GM	PC^2^	1.41	0.5 (0.41, 0.6)	0.94 (0.84, 0.99)	0.64 (0.56, 0.72)
	Cortical Summary	1.29	0.41 (0.32, 0.5)	0.92 (0.81, 0.98)	0.57 (0.49, 0.65)
Neuritic plaque					
WM	PC^2^	0.77	0.75 (0.66, 0.83)	0.84 (0.72, 0.92)	0.78 (0.71, 0.84)
	Cortical Summary	0.70	0.71 (0.61, 0.8)	0.84 (0.72, 0.92)	0.76 (0.68, 0.82)
Cbllm GM	PC^2^	1.34	0.65 (0.55, 0.74)	0.85 (0.74, 0.93)	0.73 (0.65, 0.79)
	Cortical Summary	1.21	0.65 (0.55, 0.74)	0.8 (0.68, 0.89)	0.71 (0.63, 0.78)

Numbers in parentheses represent 95% confidence intervals. Abbreviations: PC^2^ = posterior cingulate and precuneus; cortical summary = a composite cortical summary region composed of frontal, anterior/posterior cingulate, lateral parietal, and lateral temporal regions; WM = eroded white matter; Cbllm GM = cerebellar gray matter

For determining neuritic plaque positivity, the same SUVR cutoff of 0.77 in PC^2^ + WM achieved the highest Youden index, with a sensitivity of 0.75 (95% CI 0.66–0.83) and a specificity of 0.84 (95% CI 0.72–0.92). cortical summary + WM achieved the highest Youden index at an SUVR cutoff of 0.70, with sensitivity of 0.71 (95% CI 0.61–0.8) and specificity of 0.84 (95% CI 0.72–0.92). When using cerebellar gray matter as reference, PC^2^ + Cbllm GM achieved the highest Youden index at a SUVR cutoff of 1.34, with sensitivity of 0.65 (95% CI 0.55–0.74) and specificity of 0.85 (95% CI 0.74–0.93). cortical summary + Cbllm GM achieved Youden index at a SUVR cutoff of 1.21, with sensitivity of 0.65 (95% CI 0.55–0.74) and specificity of 0.8 (95% CI 0.68–0.89).

#### Discrepancy between PET- and pathology-based classifications

Because temporal differences between amyloid PET and amyloid neuropathology could contribute to discrepancies between PET and postmortem measures, we examined participants whose classifications were discrepant with neuropathology based on the optimal cutoff of 0.77 in the best performing combination of PC^2^ + WM and show the characteristics of participants with concordant and discordant PET prediction and neuropathology. For amyloid beta positivity, there were 39 participants with discrepant PC^2^ + WM prediction and pathology, of which 32 (82%) were positive on postmortem neuropathology but negative on PC^2^ + WM prediction; among these, 11 (34%) had an A-score of 2. Conversely, there were only 7 participants who were negative on postmortem neuropathology but positive on PC^2^ + WM prediction, and of these 6 (86%) had an A-score of (1) For neuritic plaque positivity, of 36 total discordant cases, 26 participants (72%) were positive on postmortem neuropathology but negative on PC^2^ + WM prediction of which 15 (58%) had a C-score of (2) Among the 10 participants who were negative on postmortem neuropathology but positive on PC^2^ + WM prediction, 6 (60%) had a C-score of 1. When comparing participant characteristics between concordant and discrepant groups we found no major differences in age at PET, age at death, sex, or education between the two groups. We additionally examined the interval between PET imaging and death (Supplementary Fig. 7), where concordant cases and discrepant cases showed similar distributions.

### Sensitivity analyses

In models adjusting for demographic factors and APOE genotype, the predictive performance of SUVRs remained robust with almost identical results to the models without demographic adjustment (Supplementary Fig. 3). For both amyloid beta positivity and neuritic plaque positivity, the two SUVRs using the WM reference region demonstrated better performance, with PC^2^ + WM achieving the highest AUC values (0.85 for amyloid beta positivity and 0.84 for neuritic plaque positivity).

In models that further accounted for the time interval from PET scan to death (Supplementary Fig. 4), PC^2^ + WM continued to yield the highest AUC values (0.85 for amyloid beta positivity and 0.83 for neuritic plaque positivity). In addition, we conducted subgroup analyses (using the SUVR only model) among 137 participants who died within five years and 85 participants who died within three years following PET imaging. In both subgroups, PC^2^ + WM consistently remained the best-performing SUVR (Supplementary Fig. 5). Specifically, in the subgroup who died within five years following PET, PC^2^ + WM achieved AUC values of 0.84 for amyloid beta positivity and 0.81 for neuritic plaque positivity. In the subgroup restricted to participants who died within three years from PET, PC^2^ + WM maintained high predictive performance, with AUC values of 0.84 for amyloid beta positivity and 0.82 for neuritic plaque positivity.

Lastly, when using the alternative dichotomization definitions for amyloid beta positivity (A3) and neuritic plaque positivity (C3) PC^2^ + WM continued to demonstrate the highest predictive performance, achieving AUC values of 0.8 for amyloid beta positivity and 0.85 for neuritic plaque positivity (Supplementary Fig. 6).

## Discussion

In this study, we evaluated the predictive performance of four SUVR calculations for amyloid beta positivity and neuritic plaque positivity, using ROC analyses and accompanying sensitivity analyses. The two SUVRs using white matter as the reference region consistently demonstrated superior performance compared to the two using cerebellar gray matter. Additionally, the combination of posterior cingulate and precuneus as the ROI and white matter as the reference region (PC^2^ + WM) was the best performing SUVR, achieving the highest AUC values for predicting both amyloid beta positivity (AUC = 0.84) and neuritic plaque positivity (AUC = 0.82). This finding stayed consistent in sensitivity analyses that adjusted for covariates, including sex, age at PET, education level, and the time interval from PET to death, as well as in restricted cohorts of participants who underwent PET imaging proximate to death. Using the best performing SUVR, we identified an optimal cutoff of 0.77 for PC^2^ + WM for predicting both amyloid beta positivity and neuritic plaque positivity. Cases that were discrepant between amyloid PET and pathology tended to be on the borderline (either the category above or below the cutoff) and tended to be negative on PET prediction and positive on amyloid pathology.

The primary purpose of this study was to evaluate the performance of appropriate regions for the quantification of amyloid burden on PET in the oldest-old. Neuropathology, as the gold standard for amyloid pathology, allows us to evaluate the accuracy of SUVRs in distinguishing amyloid-positive from amyloid-negative cases. This study is unique due to its large sample size of individuals with both amyloid PET imaging and autopsy data from the oldest-old age group, an important age group where dementia and AD neuropathology are most prevalent. With PET imaging as the best biomarker currently available for diagnosing Alzheimer’s disease during life, these findings offer critical evidence for assigning treatment based on amyloid positivity. Specifically, our study suggests the PC^2^ + WM is a robust SUVR calculation method with respect to both examined neuropathological outcomes for amyloid accumulation, which could be consequential for clinical decision-making.

Our finding that the posterior cingulate and precuneus, when normalized to white matter, provided the highest accuracy for amyloid quantification aligns with several previous studies. The posterior cingulate was previously identified as a site for early amyloid accumulation in AD [37]. In addition, posterior cingulate and precuneus were most closely associated with cognitive status and provided the highest sensitivity and specificity for amyloid status at autopsy [21, 28]. The use of white matter as a reference region has been shown to improve the detection of amyloid changes [7, 9, 13, 15]. Thus, some of the results from the current study may be related to longitudinal progression of amyloid, as some previous studies have noted that WM reference better corresponds with amyloid accumulation [13, 15]. For instance, Landau et al. reported that the use of the subcortical white matter as a reference region was more accurate in detecting amyloid beta changes than the use of the cerebellum or pons [13] and Chen et al. demonstrated that a white matter reference region can improve the statistical power for characterizing longitudinal amyloid beta PET changes [15]. In the study by Chiao et al., WM and/or pons reference, as well as more limited cortical regions such as the anterior cingulate and posterior cingulate cortex, resulted in larger effect size estimates of amyloid removal in data from the Phase 1b PRIME Study of Aducanumab [7]. Thus, while standard sets of cortical composite regions are generally used for the reporting of standardized amyloid burden on PET through centiloids, our results and previous studies highlight benefits of using white matter as a reference region and the good performance of certain cortical regions. Furthermore, our study highlights that these points may be more salient in the oldest-old, and thus studies and clinical trials focusing on this important population should consider using WM reference and more limited cortical regions alongside more routine measures of amyloid PET burden generally used in younger cohorts.

We found that using the WM reference consistently yielded better predictive performance as compared to the cerebellum gray matter. Although the difference in ROC curves between PC^2^ and the cortical summary did not reach statistical significance when using the WM reference, the AUC values consistently favored the PC^2^. And, when using the cerebellar GM reference, PC^2^ performed significantly better than the cortical summary region. The superior performance of PC^2^ may have been less pronounced in some of our analyses because WM was a better reference region than cerebellar GM which resulted in a narrowed performance gap between the target regions. Additionally, the cases that were discrepant between PET prediction and neuropathology tended to be negative on PET and positive on pathology but in the intermediate range (A-score and C-scores of 2 instead of 3), which could be related to additional amyloid accumulation in the time between the scan and autopsy.

The pipeline implemented in this study is similar to the MRI-free approach discussed in previous studies, and is a particularly useful approach as analysis of PET data without relying on MRI may be necessary for both practical and clinical reasons [27]. First, requiring MRI introduces additional costs and logistical complexities, including extended scheduling and the need for specialized equipment and expertise. Second, many older adults or individuals with pacemakers, metallic implants, or severe claustrophobia cannot safely undergo MRI, thereby limiting study participation and representativeness. Therefore, developing reliable SUVR measures that quantify amyloid burden that are MRI independent can reduce study costs, simplify protocols, and promote broader inclusion. Additionally, while current approved anti-amyloid therapies require extensive MRI follow-up due to amyloid related imaging abnormalities (ARIA) [38, 39], there are still participants who may be unable to undergo MRI due to complications [40, 41], and future treatments for amyloid that are less reliant on MRI or treatment schemes that depend on ruling out amyloid may benefit from a robust MRI-free amyloid PET pipeline such as the one presented in this study.

### Strengths, limitations, and future directions

Our study has several strengths. First, we had a large cohort of 165 participants who underwent both PET imaging and postmortem autopsy. Sample sizes from previous amyloid quantification studies that had neuropathology data ranged from 42 to 68 individuals [42–44]. In addition, our amyloid PET quantification methods were specifically developed with consideration of age-related issues, such as increased cortical atrophy, which ensures more accurate amyloid assessment in the oldest-old population (90 + years)—a rapidly expanding demographic in neuroimaging research. We also performed a series of sensitivity analyses to probe the robustness of our findings.

Our study also has some limitations. First, although we have a relatively large cohort compared with previous studies, 48.5% of the participants in the study had a PET scan more than 3 years before autopsy. Although we controlled for age at PET in sensitivity analyses, the prolonged interval in some cases opens the possibility that amyloid beta pathology may have evolved to a considerable degree after PET scan, potentially reducing sensitivity and specificity for the SUVRs. Nonetheless, our analyses found almost identical results when limiting analyses to within 3 years of death and found no significant interaction between amyloid PET burden and the interval to death. Our study predominantly involved individuals of white race/ethnicity and high education, which may limit the generalizability of our findings. Future research that includes more diverse populations may further corroborate our findings. Another limitation in our study is that while our SUVR measures reflect amyloid burden at specific regions of the brain, such as posterior cingulate and precuneus, the postmortem neuropathological assessments involve sampling amyloid at specific regions in the brain and assigning amyloid load throughout the brain. Future studies incorporating region-specific postmortem quantification, or more precise imaging-pathology co-registration, involving these same regions may help clarify the sources of discrepancy between PET and neuropathologic measures. Finally, we utilized an MRI free pipeline due to lacking MRIs for certain participants and because MRIs are often contraindicated in the oldest-old (e.g. because of pacemakers). However, further analyses using both MRI and PET scans could provide a better picture of the performance of regional amyloid SUVRs and could additionally correct for issues of atrophy through the use of partial volume correction techniques [45, 46].

### Conclusion

Overall, this study found that using a combined posterior cingulate and precuneus region normalized to white matter as a reference region for amyloid PET SUVR quantification provided the highest predictive performance for amyloid pathology in a group of oldest-old participants. By identifying an optimal SUVR cutoff, our findings offer clinicians and researchers a potentially more reliable and practical biomarker for accurately detecting amyloid pathology in this important age group. Given the rapid growth and clinical importance of the oldest-old population, these results hold significant implications for the diagnosis, management, and future research of Alzheimer’s disease and related dementias in advanced age.

## Supplementary Information

Below is the link to the electronic supplementary material.

Supplementary Material 1

## Data Availability

No datasets were generated or analysed during the current study.
