# Supplementary material for: Amyloid quantification in the oldest-old: selecting regions for optimizing correspondence between postmortem pathology and amyloid PET
Source: Acta Neuropathol Commun. 2025 Dec 1;14:7. doi: 10.1186/s40478-025-02198-3 (PMC12777254; doi:10.1186/s40478-025-02198-3)

Supplementary Figures:

**Supplementary Fig. 1** Histogram of time between PET imaging and death


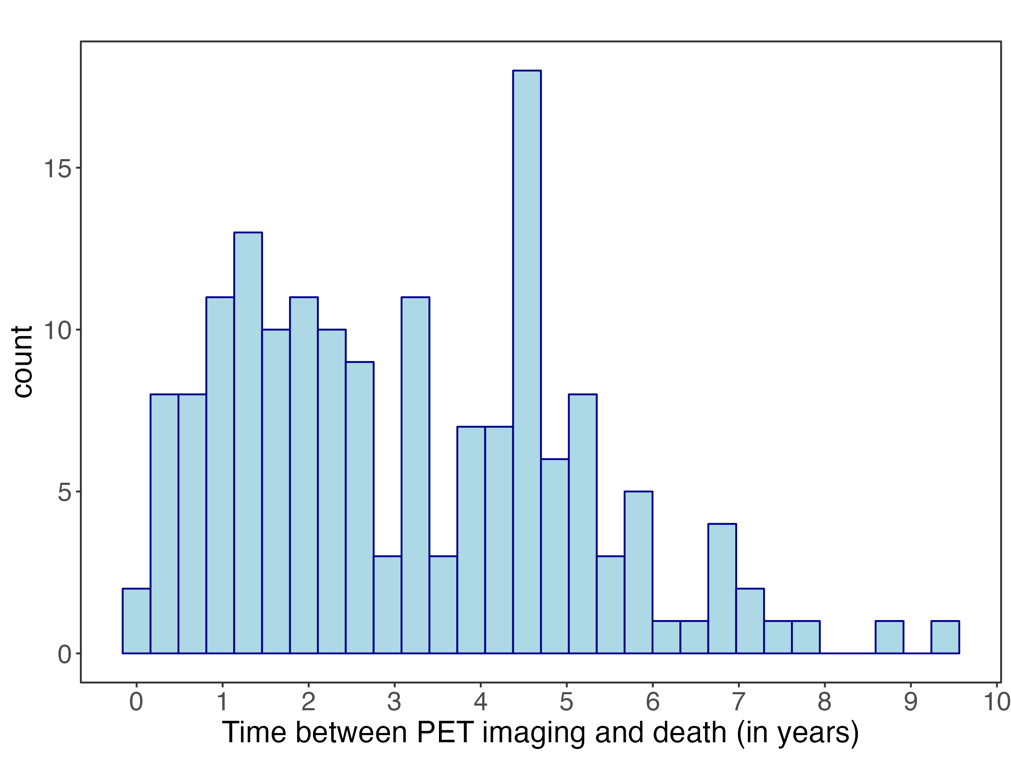


**Supplementary Fig. 2** Correlations between the four SUVRs. Each cell represents the correlation between two SUVRs, and cell with more red coloring indicates higher correlation. Abbreviations: PC^2^ = posterior cingulate and precuneus; WM = eroded subcortical white matter; Cbllm GM = cerebellar gray matter


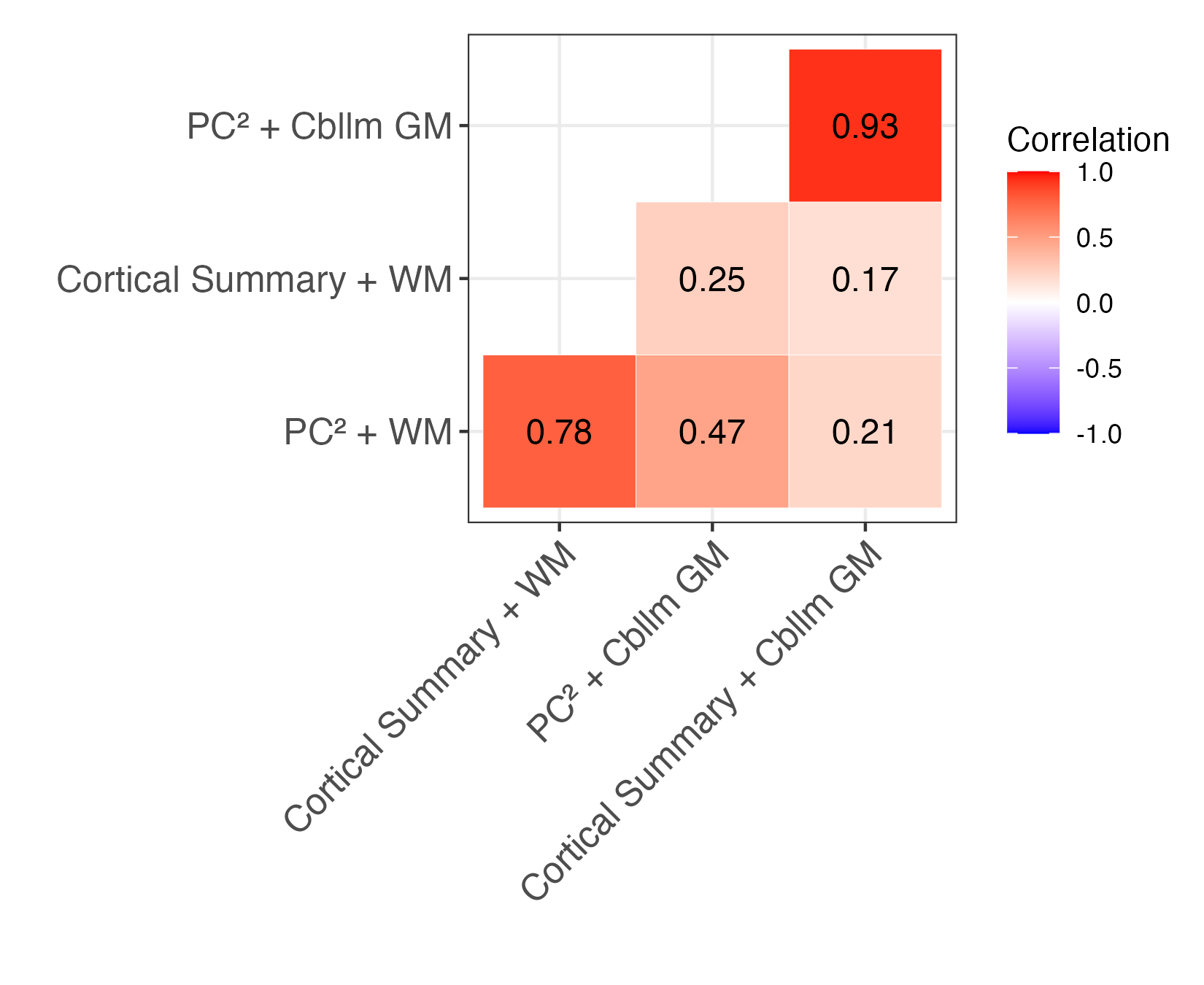


**Supplementary Fig. 3** ROC curves for amyloid beta positivity, and neuritic plaque positivity prediction adjusted for sex, age at PET, education, and APOE genotype. After adjusting for demographic factors and APOE genotype, PC^2^ + WM remained the best SUVR for predicting both amyloid outcomes. The numbers in parentheses represent 95% confidence intervals. Abbreviations: PC^2^ = posterior cingulate and precuneus; WM = eroded white matter; Cbllm GM = cerebellar gray matter


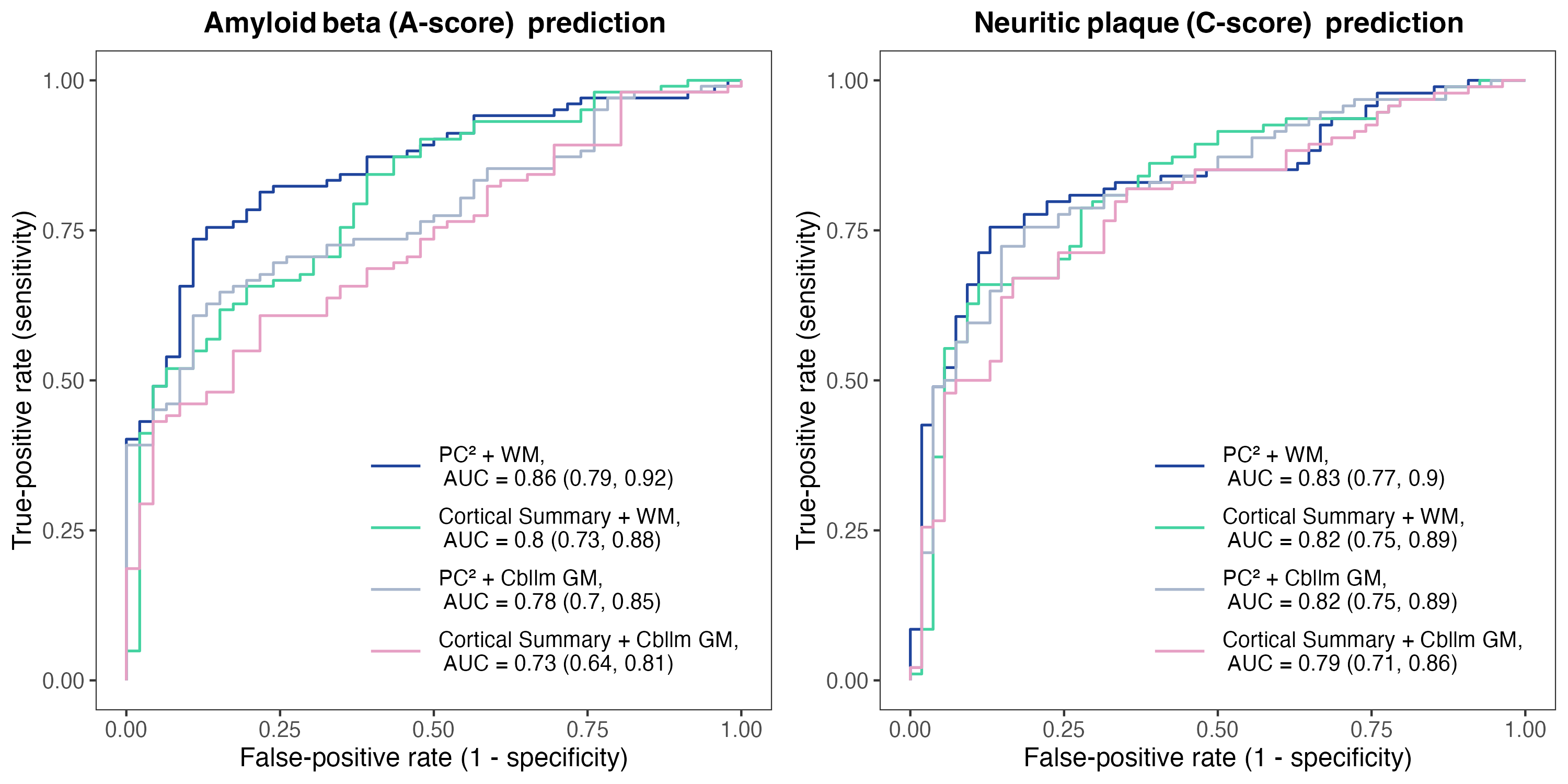


**Supplementary Fig. 4** ROC curves for amyloid beta and neuritic plaque positivity prediction adjusted for sex, age at PET, education level, time interval between PET and death and the interaction of SUVR and time interval between PET and death. In this sensitivity analysis, PC² + WM continued to yield the highest AUC values. The numbers in parentheses represent 95% confidence intervals. Abbreviations: PC^2^ = posterior cingulate and precuneus; WM = eroded white matter; Cbllm GM = cerebellar gray matter


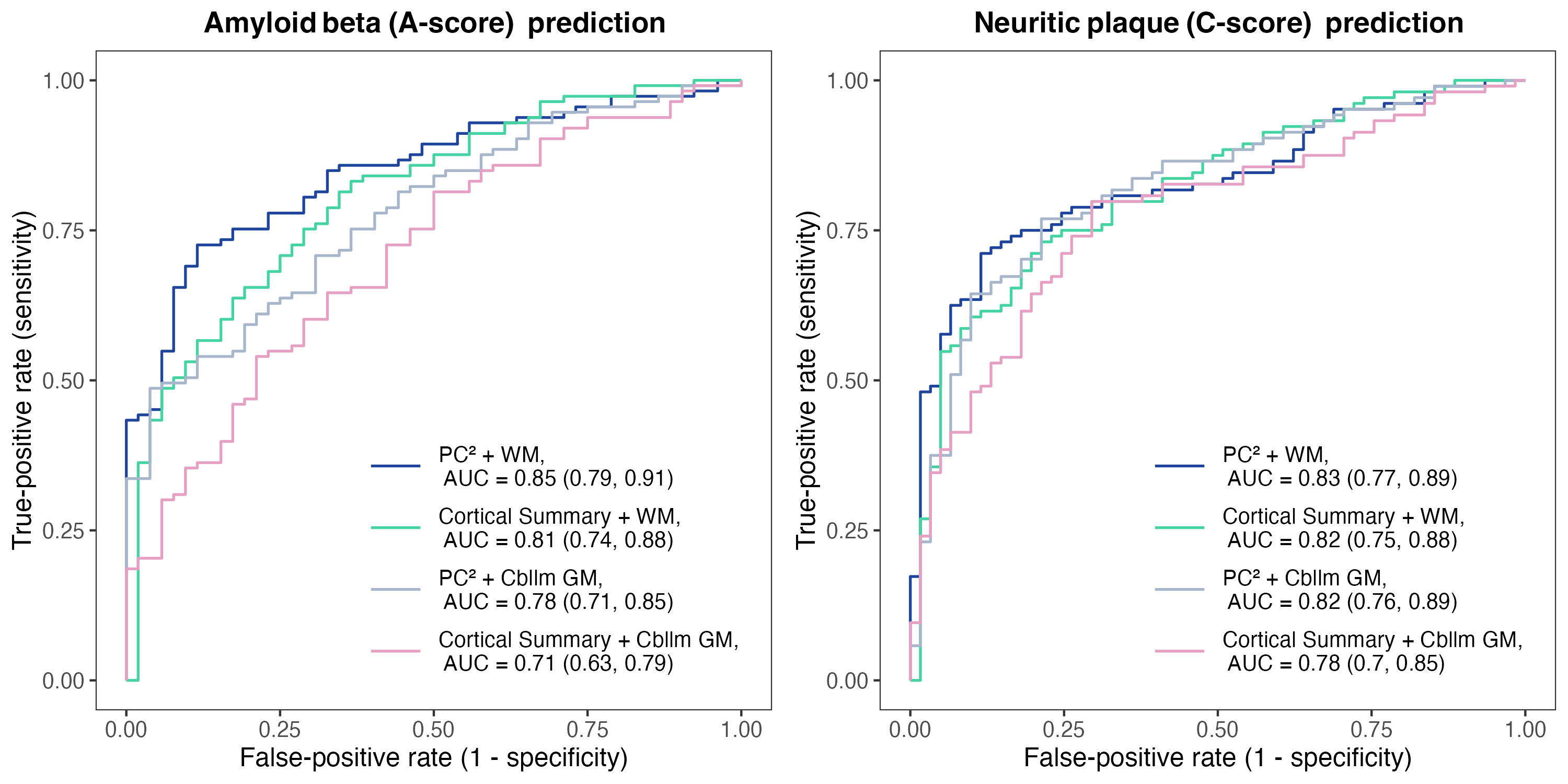


**Supplementary Fig. 5** ROC curves for amyloid beta and neuritic plaque positivity prediction in two restricted samples: five years between PET imaging and death (panel A and panel B) and three years between PET imaging and death (panel C and panel D). The numbers in parentheses represent 95% confidence interval. Abbreviations: PC^2^ = posterior cingulate and precuneus; WM = eroded white matter; Cbllm GM = cerebellar gray matter


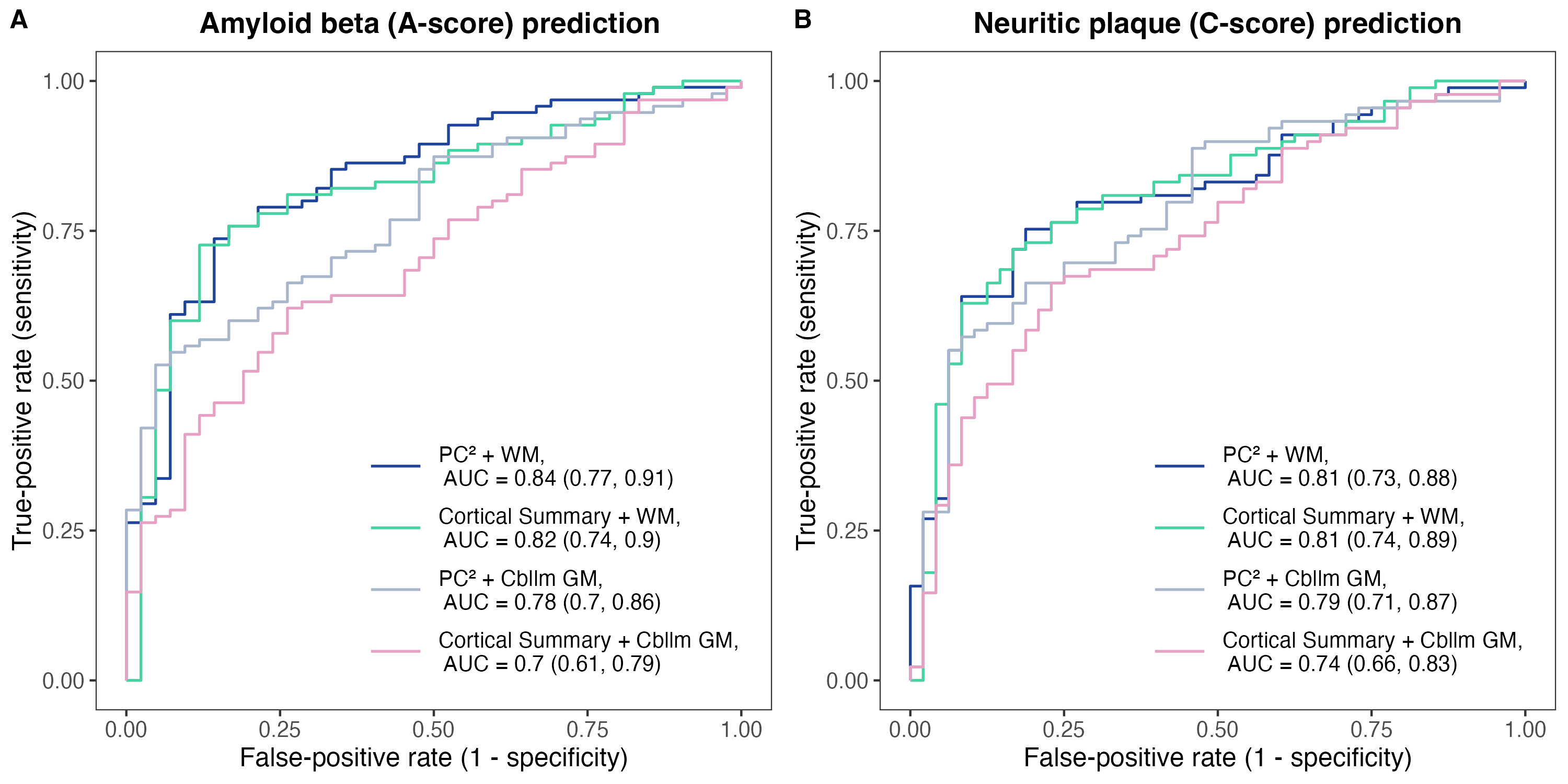


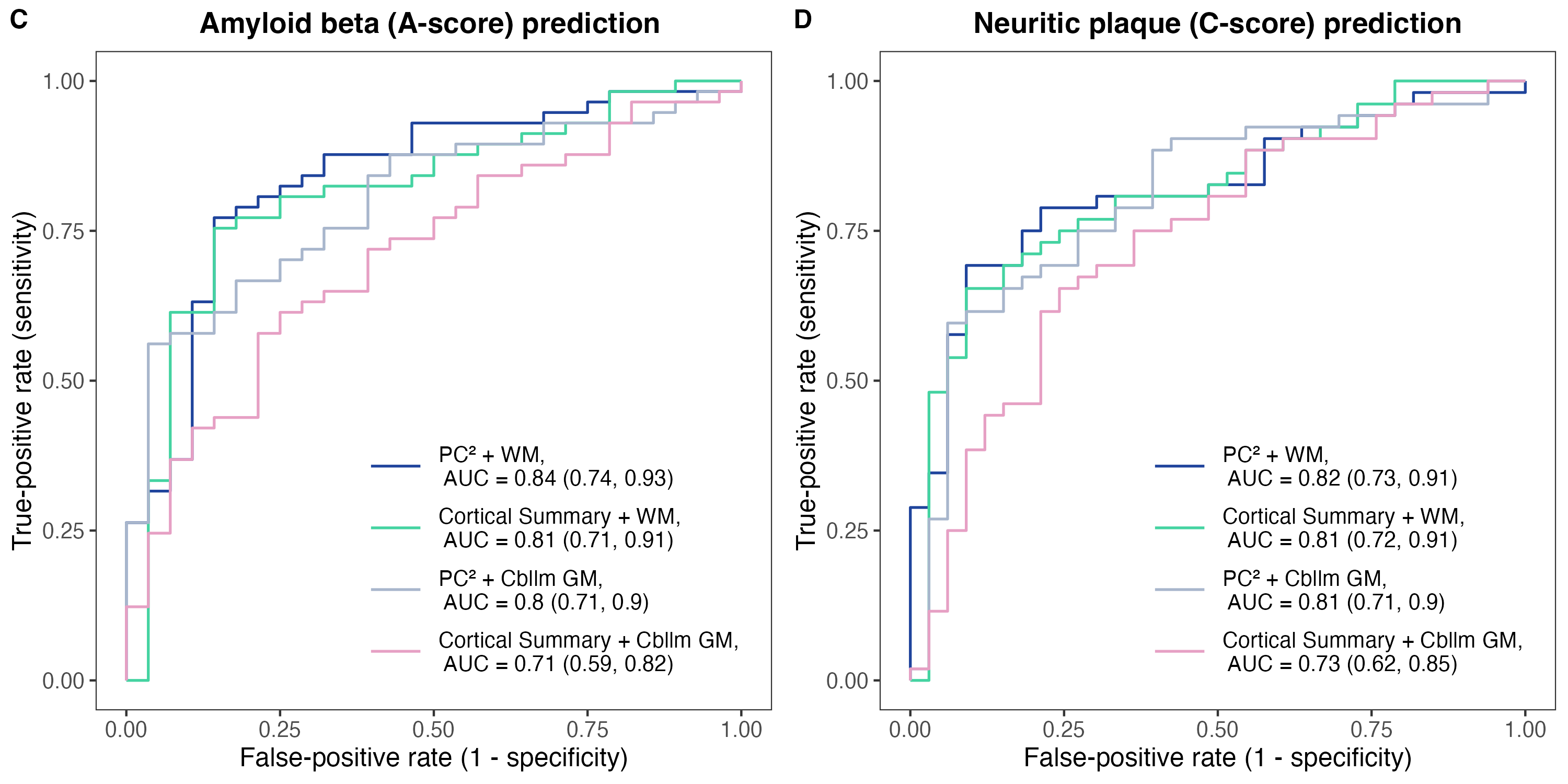


**Supplementary Fig. 6** ROC curves for amyloid beta and neuritic plaque positivity prediction under a different way of dichotomizing amyloid positivity specifically (A) amyloid beta positivity was defined as A3 and (B) neuritic plaque positivity was defined as C3. The numbers in parentheses represent 95% confidence interval. Abbreviations: PC^2^ = posterior cingulate and precuneus; WM = eroded white matter; Cbllm GM = cerebellar gray matter


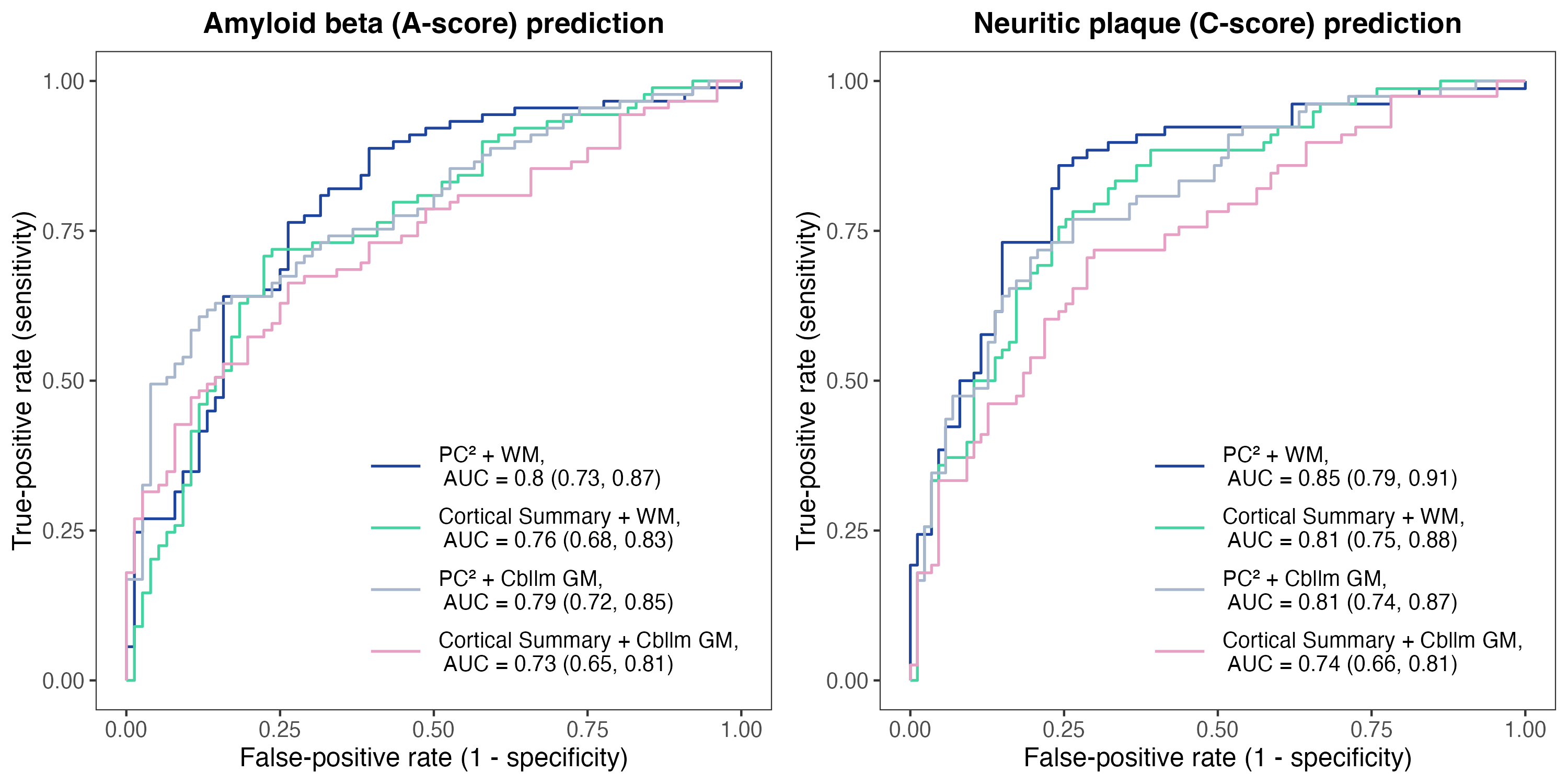


| **Supplementary Table 1** Characteristics of the participants stratified by concordant and discrepant cases. | | | | | | | | |
| --- | --- | --- | --- | --- | --- | --- | --- | --- |
|  | |  | **Amyloid beta positivity**  **_______________________________** | | | **Neuritic plaque positivity**  **______________________________** | | |
|  |  | Whole group  (N = 165) | Concordant  (N = 126) | Discrepant  (N = 39) | *P*-value | Concordant  (N = 129) | Discrepant  (N = 36) | *P*-value |
| Age at PET | | 94.23 (3.20) | 94.32 (3.35) | 93.91 (2.66) | 0.7 | 94.40 (3.39) | 93.59 (2.30) | 0.4 |
| Age at death | | 97.4 (3.6) | 97.4 (3.5) | 97.4 (3.8) | 0.9 | 97.6 (3.7) | 96.7 (3.3) | 0.3 |
| PET–death interval (years) | | 3.14 (1.97) | 3.03 (1.92) | 3.48 (2.09) | 0.2 | 3.16 (2.07) | 3.07 (1.58) | 0.8 |
| Female | | 98 (59%) | 75 (60%) | 23 (59%) | >0.9 | 77 (60%) | 21 (58%) | 0.9 |
| College graduate | | 90 (55%) | 70 (56%) | 20 (51%) | 0.6 | 74 (57%) | 16 (44%) | 0.2 |
| APOE $\varepsilon4$+^a^ | | 24 (16%) | 21 (19%) | 3 (8.6%) | 0.2 | 20 (17%) | 4 (13%) | 0.6 |
| A-score | 0 | 16 (9.7%) | 15 (12%) | 1 (2.6%) | 0.022 |  |  |  |
|  | 1 | 36 (22%) | 30 (24%) | 6 (15%) |  |  |  |  |
|  | 2 | 24 (15%) | 13 (10%) | 11 (28%) |  |  |  |  |
|  | 3 | 89 (54%) | 68 (54%) | 21 (54%) |  |  |  |  |
| C-score | 0 | 36 (22%) |  |  |  | 32 (25%) | 4 (11%) | <0.001 |
|  | 1 | 25 (15%) |  |  |  | 19 (15%) | 6 (17%) |  |
|  | 2 | 26 (16%) |  |  |  | 11 (8.5%) | 15 (42%) |  |
|  | 3 | 78 (47%) |  |  |  | 67 (52%) | 11 (31%) |  |
| *“Concordant” represents cases where amyloid status determined by PC² + WM SUVR prediction agreed with postmortem autopsy findings, while “discrepant” represents cases where the two measures disagreed. An A score of 2 or 3 (A2 or A3) is defined as amyloid beta positive; A C score of 2 or 3 (C2 or C3) is defined as neuritic positive. P-values were obtained from t-test or Fisher’s exact test.*  *APOE* $\varepsilon4$*+^a^ 17 participants did not have available APOE ε4 data* | | | | | | | | |

**Supplementary Fig. 7** Histogram of time between PET imaging and death, colored by whether the cutoff of 0.78 in PC² + WM predict postmortem amyloid beta positivity (on the left) and neuritic plaque positivity (on the right). The blue bars represent the concordant cases where amyloid status determined by SUVR prediction agreed with postmortem autopsy findings. The red bars represent the discrepant cases where the two measures disagreed.


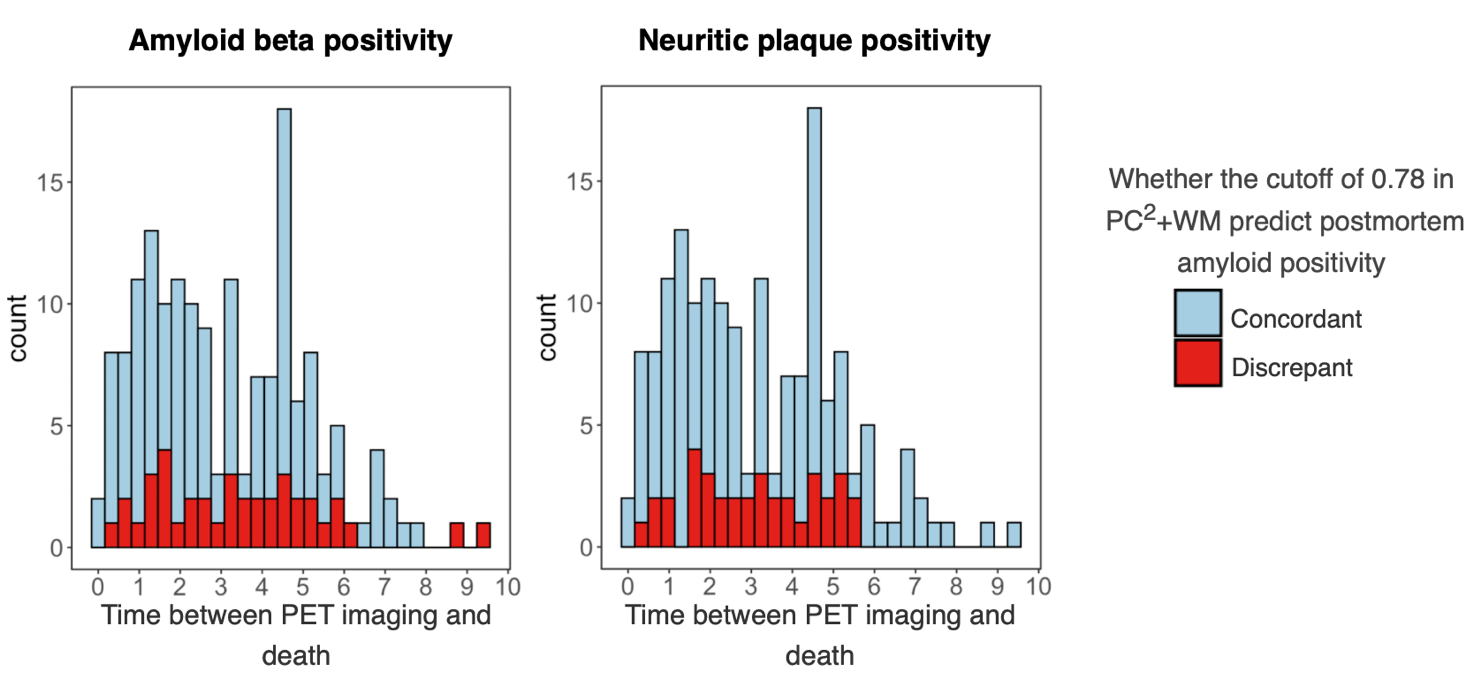

Supplement: Supplementary file 1 — Supplementary Material 1 [file 40478_2025_2198_MOESM1_ESM.docx]
